# A simple dosimetric approach to spatially fractionated GRID radiation therapy using the multileaf collimator for treatment of breast cancers in the prone position

**DOI:** 10.1002/acm2.13040

**Published:** 2020-10-29

**Authors:** Natasha L. Murphy, Rino Philip, Matt Wozniak, Brian H. Lee, Eric D. Donnelly, Hualin Zhang

**Affiliations:** ^1^ Department of Radiation Oncology Robert H. Lurie Comprehensive Cancer Center Northwestern University Feinberg School of Medicine Northwestern Memorial Hospital Chicago IL 60611 USA

**Keywords:** breast cancer, GRID therapy, spatially fractionated radiation therapy, therapeutic ratio

## Abstract

The purpose of this study was to explore the treatment planning methods of spatially fractionated radiation therapy (SFRT), commonly referred to as GRID therapy, in the treatment of breast cancer patients using multileaf collimator (MLC) in the prone position. A total of 12 patients with either left or right breast cancer were retrospectively chosen. The computed tomography (CT) images taken for the whole breast external beam radiation therapy (WB‐EBRT) were used for GRID therapy planning. Each GRID plan was made by using two portals and each portal had two fields with 1‐cm aperture size. The dose prescription point was placed at the center of the target volume, and a dose of 20 Gy with 6‐MV beams was prescribed. Dose‐volume histogram (DVH) curves were generated to evaluate dosimetric properties. A modified linear‐quadratic (MLQ) radiobiological response model was used to assess the equivalent uniform doses (EUD) and therapeutic ratios (TRs) of all GRID plans. The DVH curves indicated that these MLC‐based GRID therapy plans can deliver heterogeneous dose distribution in the target volume as seen with the conventional cerrobend GRID block. The plans generated by the MLC technique also demonstrated the advantage for accommodating different target shapes, sparing normal structures, and reporting dose metrics to the targets and the organs at risks. All GRID plans showed to have similar dosimetric parameters, implying the plans can be made in a consistent quality regardless of the shape of the target and the size of volume. The mean dose of lung and heart were respectively below 0.6 and 0.7 Gy. When the size of aperture is increased from 1 to 2 cm, the EUD and TR became smaller, but the peak/valley dose ratio (PVDR) became greater. The dosimetric approach of this study was proven to be simple, practical and easy to be implemented in clinic.

## 1 INTRODUCTION

Clinical results have indicated that megavoltage spatially fractionated radiation therapy (SFRT, or simply called GRID therapy hereafter) provided by modern linear accelerator machines can significantly improve the therapeutic window in the treatment of bulky tumors.^1^, ^2^, ^3^, ^4^, ^5^ Researchers have attributed the therapeutic advantages identified in the GRID radiation field to the bystander effect, which is stronger in the high gradient field^6^; although the underlying reasons for improved responses can be explained by other mechanisms,^7^ in which the potential therapeutic advantage of GRID therapy was derived from the radiobiological modeling results based on the different radio‐sensitivities of normal and cancerous cells in the target volume. As reported by Zwicker et al.^8^ and Zhang et al.,^9^, ^10^ in theory the GRID therapy takes advantage of the fact that normal cells interspersed in the cancerous cells in the target volume in general have superior repair capabilities over cancer cells. When normal tissue cells are spared by GRID therapy in low dose zones, those lower‐irradiated areas can serve as centers of regrowth for normal tissues. In the high dose zones, however, there will be an intensive killing of cancer cells and normal cells as well, consequently the communications between the cancer cells are obstructed throughout the tumor volume. With the spatially fractionated radiation fields, the cancer cell killing rate is maintained, whereas the normal cell survival is increased due to the existence of the cold zones and normal cell repair, thereby providing a clinical advantage shown by the tumor shrinkage and increased radiation tolerance. The latest study by Zhang et al.^7^ demonstrated that GRID therapy provided a pronounced therapeutic advantage in both hypofractionated and traditionally fractionated regimens as compared with the results seen with single‐fraction, open debulking field regimens. However, from the radiobiological modeling results, the true therapeutic advantage (after separating the benefit of fractionation) exists only in hypofractionated GRID therapy.^7^ Of note, clinical outcomes and theoretical studies have indicated that a course of open‐field radiotherapy is needed to further control tumor growth after a large‐fraction dose with GRID therapy as the equivalent uniform dose (EUD) of GRID therapy is significantly less than standard prescription doses.^1^, ^3^, ^4^, ^7^, ^10^

Clinical trials of GRID therapy continue adding useful data each year and patients with bulky cancer have shown benefit from this unique treatment; yet researchers are still striving for better techniques to deliver GRID therapy. One effort is avoiding using heavy and inconvenient Cerobend GRID block collimators which are not able to take into account tumor shape and normal structure sparing.^11^, ^12^ New GRID therapy approaches, which use the existing technology of tomotherapy,^12^ or high precision multileaf collimators (MLCs)^11^, ^13^, ^14^, ^15^ or stereotactic radiotherapy apparatus^16^ found in most modern radiotherapy linear accelerator machines, are expected to facilitate the use of GRID therapy in the radiation oncology clinic. It has been shown that such techniques can alleviate the work intensity and collateral damage concerns of the large dose impact of GRID therapy to the adjacent organs.^14^

In this study, the GRID therapy plans were developed by utilizing a treatment planning system (TPS) associated with an MLC capable linear accelerator machine for breast cancer patients in the prone position. The prone position was chosen because the supine position poses a potential concern for increased lung and heart dose.^17^ It has been reported that the prone position can significantly reduce the dose to the organs at risks (OARs) in the setting of breast EBRT,^18^ thus choosing prone position for breast GRID therapy was expected to mitigate the concern of high dose streaks made by the GRID field.

## 2 MATERIALS AND METHODS

### 2.A Patient selection and treatment setup

A total of 12 patients with either left or right breast cancer, who had planning target volumes (PTV) ranging from 354 to 1778 cm^3^ (with average volume of 814.5 cm^3^) and were treated with the whole breast external beam radiation therapy (WB‐EBRT) in the prone position, were retrospectively chosen for this GRID therapy study. There were six left‐sided breast patients and six right‐sided breast patients. The reason why both the left sided and right sided and up to 12 patients were chosen is because, it is important to see if the proposed dosimetric approach can create consistent quality plans for different breast sides and different sizes of target volumes, for the purpose of clinical trial. Regions of interest included the breast volume, gross target volume (GTV), planning target volume (PTV), left lung, right lung, ribs, and heart. The whole left or right breast with a negative 0.5 cm margin was chosen as the PTV of GRID therapy, in accordance with the protocol of WB‐EBRT regimens in which the PTV is usually taken as the whole breast minus 0.5 cm from the skin to minimize skin dose in the plan. A single fraction of 20 Gy prescription dose was used in all patients of this study, the DVH curves of target volumes and OARs were calculated to show the dosimetric results achieved by the plans. The dose of 20 Gy was selected as this GRID fraction dose has been widely used in various clinics and proven to be safe and effective.^3^, ^4^, ^5^, ^14^

During the treatment, the patients were setup in the prone position using Bionix Prone Breast System (Bionix Radiation Therapy, LLC. 5154 Enterprise Blvd., Toledo, OH) with the head turned in the opposite direction of the treated breast. Both arms were positioned up in a vac‐lok^TM^ (CIVCO Radiotherapy, 2303 Jones Blvd., Coralville, Iowa) and holding on to poles. For added comfort and support, a piece of styrofoam was placed under the sternum. Daily imaging included a daily CBCT (cone beam computed tomography), and a chest wall matching was conveniently used.

### 2.B Dosimetric approach for creating MLC‐based GRID therapy plans

**Fig. 1:**
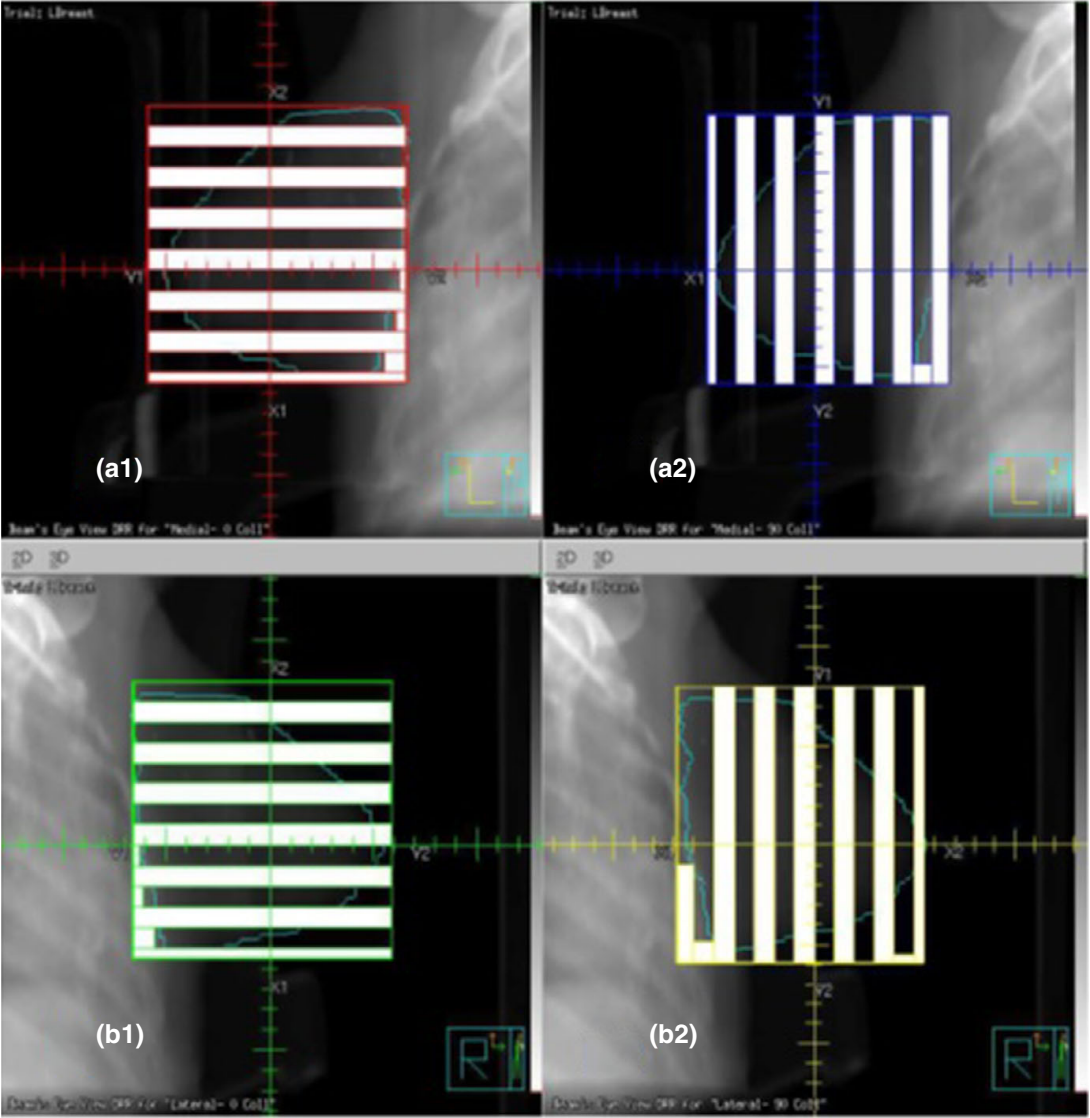
Schematic diagram of multi‐leaf collimators (MLCs) used in forming a GRID field in this study. Upper two figures (a1 and a2) show two orthogonal fence fields used to form one side of tangential GRID field. Lower two orthogonal fence fields (b1 and b2) were used for another side of the tangent GRID field.

An Elekta Infinity^TM^ clinical linear accelerator (Elekta, Stockholm, Sweden) equipped with MLCs was used in planning as the treatment delivery machine. This machine has 40 pairs of MLCs with a width of 1 cm for each leaf for beam shaping. Philips Pinnacle^3^ (Philips Radiation Oncology Systems, Fitchburg, WI) version 9.10 treatment planning software was used for planning. Two opposed tangential angles were used in each plan. The medial and lateral tangent gantry angles were selected based on patient anatomy to avoid the contralateral breast in the beams and to further minimize entry/exit dose into the heart and lung. The GRID was formed using two fields on each side of tangents, thus each plan has four fields (Fig. 1). This approach was utilized because, we discovered that if every hot spot is designed as an individual radiation field,^13^ the total treatment monitor units (or time) would be far beyond the practical range. In this simple two pairs of fields approach, in each field every other MLC was extended to the opposing collimator jaw in order to create a series of striped blocks that are 1 cm wide. The field was copied and the collimator was then rotated 90 degrees to form a second field. This process was repeated on the opposing tangent fields. The combination of the two fields per tangent created a 1 by 1 cm grid in the sagittal direction. In addition, some MLCs were closed to account for the patient’s anatomy (Fig. 1). The fields were weighted to produce a homogenous population of hot spots. Also, an initial collimator rotation of 0–20 degrees was used and designed according to patient’s anatomy to minimize MLC leakage into the heart and lung.

In a commonly used and commercially available GRID block collimator (High Dose Radiation Grid; Radiation Products Design, Albertville, MN), the aperture diameter of the GRID collimator is 0.60 cm on the upper surface and 0.85 cm on the lower surface. The center‐to‐center spacing of holes on the collimator is 1.15 cm. The aperture diameter and center‐to‐center spacing are 1.3 and 1.8 cm, respectively, as projected in the plane of isocenter.^9^ So, the aperture size of our GRID plans is close to the commercial GRID block collimator. In order to understand the dosimetric impact of the aperture size, three cases were replanned with the same approach but in a 2‐cm aperture. The plans with 1 and 2‐cm apertures were dosimetrically compared.

### 2.C Dosimetric and planning criteria

A calculation point was placed in the middle of the open GRID area (hot spot area) on or near the central axis (CAX). The plans were normalized to achieve a mean dose of the breast PTV > 10 Gy, namely aiming for a mean dose of at least 50% of the prescription dose. A low dose constraint was set as at least 95% breast volume receiving 5 Gy. The maximum dose (D 0.01 cc) was limited to 24 Gy which is 120% of the prescription dose and consistent with a study presented by Costlow et al.^11^ D50 (dose covering 50% of target volume) was controlled to be around 50% of maximum dose. The maximum rib dose was limited to 20 Gy. The lowest possible mean doses of heart and lung were sought during planning, with department standards aiming for heart mean dose below 1 Gy, ipsilateral lung V10 Gy < 35% and V5 Gy < 50%.

Another calculation point was set at the center of the GTV, in order to make sure a GRID hot spot was arranged at this point, in case the point is not onto the CAX. The dose at the GTV center reaches the prescription dose 20 Gy.

### 2.D Peak/valley dose ratio (PVDR) of MLC‐based GRID therapy plan

Traditional Cerrobend‐based GRID block field has a clear peak/valley dose ratio,^9^ but for MLC‐based 3D GRID therapy, a single point of peak dose and single point of valley dose might be misrepresenting the true spatial dosimetric modulation of a GRID plan. A term of D10/D90, a ratio between the dose covering 10% of target volume and dose covering 90% of target volume was recommended by this study to represent the peak/valley dose ratio.

(1) $$PVDR=\frac{D_{10}}{D_{90}}$$

The PVDR for all plans were calculated.

### 2.E Radiobiology characteristics of GRID therapy plans

Using the dose distributions calculated by the TPS, the survival statistics of cancer and normal tissue cells inside the target volume (left or right breast PTVs) were estimated with the modified linear‐quadratic (MLQ) radioresponse model, as were the equivalent uniform doses (EUDs) and therapeutic ratio (TR) for all GRID therapy plans. Because the traditional liner quadratic (LQ) model has been found to overestimate the cell death at high doses and is only considered accurate for the dose below 10 Gy,^19^, ^20^ as a result this study uses the MLQ model proposed by Guerrero and Li,^21^ since the prescription dose of GRID plans is 20 Gy. The MLQ model introduced a shift factor δ in the LQ model to further increase survival at high dose. This model has been found to more closely predict the radiobiological responses to large dose sizes.^19^

The equation of MLQ model is as follows,

(2) $$SF_{i}=\exp\left(‐\alpha \times D_{i}‐\beta \times G\left(\lambda \times T+\delta \times D_{i}\right)\times D_{i}^{2}\right)$$

*SF_i_* is the survival fraction at the dose D_i._ α and β are radiosensitivity parameters of the cell, $G\left(\lambda T\right)=\frac{2\left(\lambda T+e^{‐\lambda T}‐1\right)}{{\left(\lambda T\right)}^{2}}$, λ is the repair rate $\left(T_{1/2}=\frac{ln2}{\lambda }\right)$, T_1/2_ is cell half‐life repair time, T is the treatment delivery time.

In 1989, Fowler^22^ suggested an α/β ratio of around 4 Gy as an approximation of breast cancer’s radiobiological response. In 2003 Guerrero and Li^21^ found some breast cancer cells still have an α/β of 10 Gy with an α of 0.3 Gy^−1^, a β of 0.03 Gy^−2^, and a half‐life repair time of 1 h. In 2017 Schwid et al^23^ found the breast cancer treatment can benefit from a nonuniform dose field radiotherapy regardless of their radiosensitivity. In this study, both of these breast cancer cell lines (α/β = 4 and 10 Gy) were used and respectively named as the acutely responding breast cancer (C1, α/β = 10 Gy) and the slowly responding breast cancer (C2, α/β = 3.846 Gy). The reason is simply because at the same dose, a cell line with a larger α/β ratio can have a smaller survival fraction (more death) than the one with a smaller α/β ratio. For this study, T_1/2_ was set at 1 h and the shift factor δ was set at 0.15 as a standard, consistent with the findings of Guerrero and Li.^21^ The radiation delivery time T was set as 0.25 h (15 min). The MLQ parameters of cancer and normal cells used in this study are listed in the Table 1.

**Table 1:** Modified linear‐quadratic (MLQ) parameters of breast cancer Cell Lines (C1 and C2) and normal tissues (N1, N2 and N3). For normal tissue, α/β = 3.1 Gy. N1 is SF(2 Gy) = 0.3, N2 is SF(2 Gy) = 0.5, and N3 is SF(2 Gy) = 0.7.^7^ N1, N2, and N3 are called radiosensitive, moderately radiosensitive, and radioresistant normal tissue, respectively.

	Breast cancer cell		Normal tissue		
	C1	C2	N1	N2	N3
α (Gy^−1^)	0.3	0.2	0.366	0.211	0.108
β(Gy^−2^)	0.03	0.052	0.118	0.068	0.035
α/β (Gy)	10	3.846	3.1	3.1	3.1
T_1/2_ (h)	1	1	1	1	1
λ (h^−1^)	0.693	0.693	0.693	0.693	0.693
δ(Gy^−1^)	0.15	0.15	0.15	0.15	0.15

The average survival fraction $\bar{\mathrm{SF}}$ was calculated by the Eq. (3) using the MLQ parameters of Table 1.

(3) $$\bar{\mathrm{SF}}=\frac{\sum_{1=1}^{i=N}SF_{i}\times f_{i}}{100}\;\sum_{i=1}^{i=N}f_{i}=100$$

*f_i_* is the fraction of target volume receiving dose Di. The average survival fraction was then utilized to solve the MLQ Eq. (4) for deriving the equivalent uniform dose (EUD).

(4) $$\exp(‐\beta \times G\left(\lambda \times T+\delta \times \left(\mathrm{EUD}\right)\right)\times {\left(\mathrm{EUD}\right)}^{2}‐\alpha \times \left(\mathrm{EUD}\right)=\bar{\mathrm{SF}}$$

Similar biological modeling considerations were applied to the interspersed normal tissue of target volume. The average surviving fraction of normal tissue in a GRID field, ${\bar{\mathrm{SF}}}_{N}\left(\mathrm{Grid}\right)$ was calculated by using the same methodology as was used for the cancer cell line but with the normal cell MLQ parameters (N1, N2, and N3 in Table 1). The ratio between the value ${\bar{\mathrm{SF}}}_{N}\left(\mathrm{Grid}\right)$ and the surviving fraction of normal cells using the EUD, that is,$\bar{SF_{N}}\left(EUD\right)$ will define the therapeutic ratio (TR) of GRID therapy.

(5) $$TR=\frac{{\bar{\mathrm{SF}}}_{N}\left(\mathrm{grid}\right)}{{\bar{\mathrm{SF}}}_{N}\left(\mathrm{EUD}\right)}$$

Because it is implied that the GRID field and open field with the same EUD will achieve the same cancer cell killing rate, a therapeutic advantage on normal tissue sparing by the GRID field is implied if TR is >1, as the GRID therapy has spared more normal tissue. However, if TR is <1, more normal cell death in the GRID field is implied, and thus a uniform dose therapy would be preferable over GRID in this scenario.

TRs are different for normal tissue N1, N2, and N3. Because breast normal tissue is considered as radiosensitive, so we just report the TRs for radiosensitive normal tissue N1. Of the note, the TRs for moderately radiosensitive normal tissue N2 and radioresistant normal tissue N3 are significantly smaller than that of N1 tissue.^7^

Before the MLQ model was proposed, in 1997 Niemierko proposed the equation of the EUD for the nonuniform spatial distribution of clonogens and nonuniform dose field with the LQ model, in which he introduced an equation where the EUD is calculated by adjusting the reference dose survival fraction in a given volume to the varied clonogen density and different local doses.^24^ The equation is as follows:

(6) $$EUD=D_{\mathrm{ref}}\times \ln\left\{\frac{\sum_{i=1}^{N}V_{i}\times {\left(SF_{2}\right)}^{\frac{D_{i}}{D_{\mathrm{ref}}}}}{\sum_{i=1}^{N}V_{i}}\right\}/\ln\left(SF_{2}\right)$$

D_ref_ is a reference dose of 2 Gy. SF_2_ is a reference survival fraction of the specific clonogen when treated with *D_ref_. Di* is the local dose, *Vi* is the local volume. The reason why EUD was also calculated by Niemierko's model, mainly because Niemierko's equation is well known by clinicians and its accuracy when used in these dosimetric results needs to be examined.

## 3 RESULTS

### 3.A Dose‐volume histograms

**Fig. 2:**
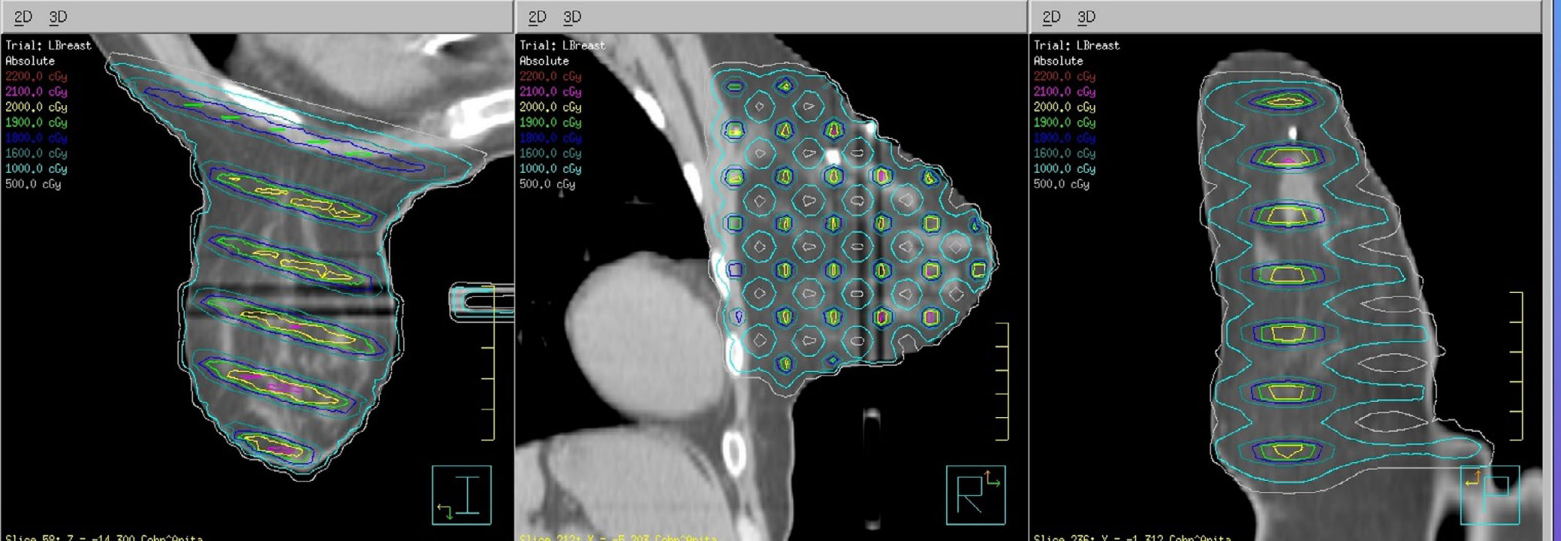
Typical isodose distribution from the corona, sagittal, and axial view. Two dark stripes shown in the breast are due to the artifacts caused by the breast support board. The mass density was changed by the artifacts from 0.9 to 0.8 g/cm^3^ in the dark strips the dosimetric impact was confirmed by treatment planning system as negligible.

Using the TPS for dosimetric planning with MLCs, six left and six right breast cancer GRID therapy plans were generated. Figure 2 shows the isodose lines of a typical breast GRID therapy plan.

**Fig. 3:**
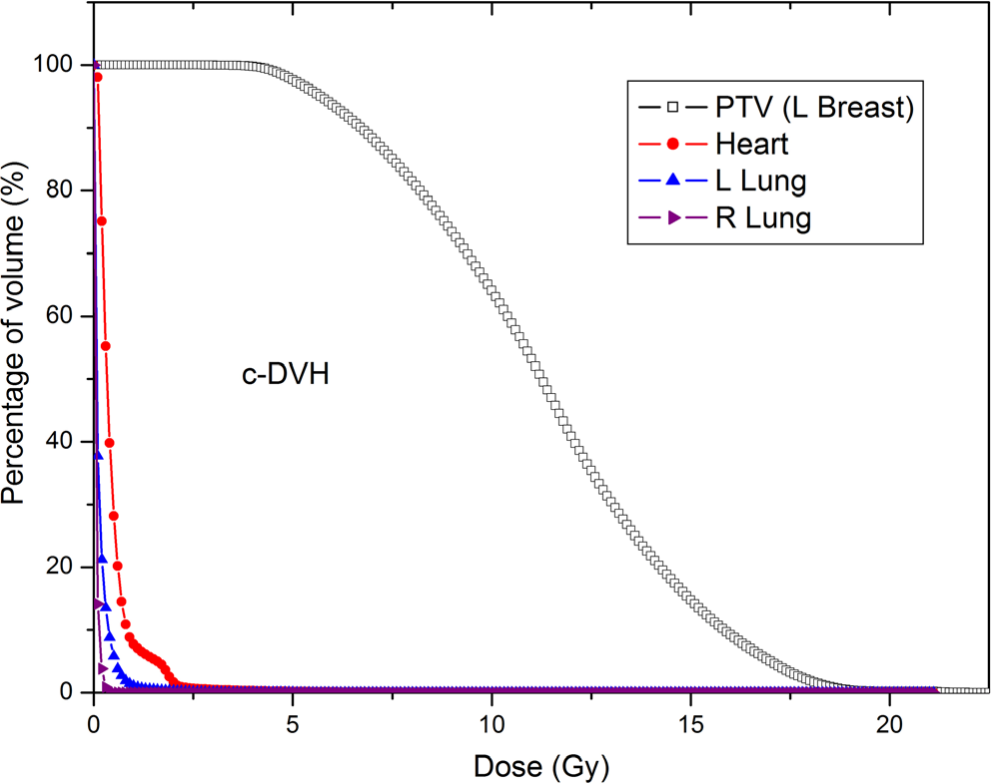
Dose‐volume histogram of a typical left breast GRID therapy plan at the prone position.

Figure 3 shows the cumulative dose‐volume histogram (c‐DVH) curves of a typical left breast GRID therapy plan. The C‐DVH curve of PTV is similar to that with the conventional Cerobend GRID block.^10^ It demonstrated that the lung and heart get very minimal dose.

**Fig. 4:**
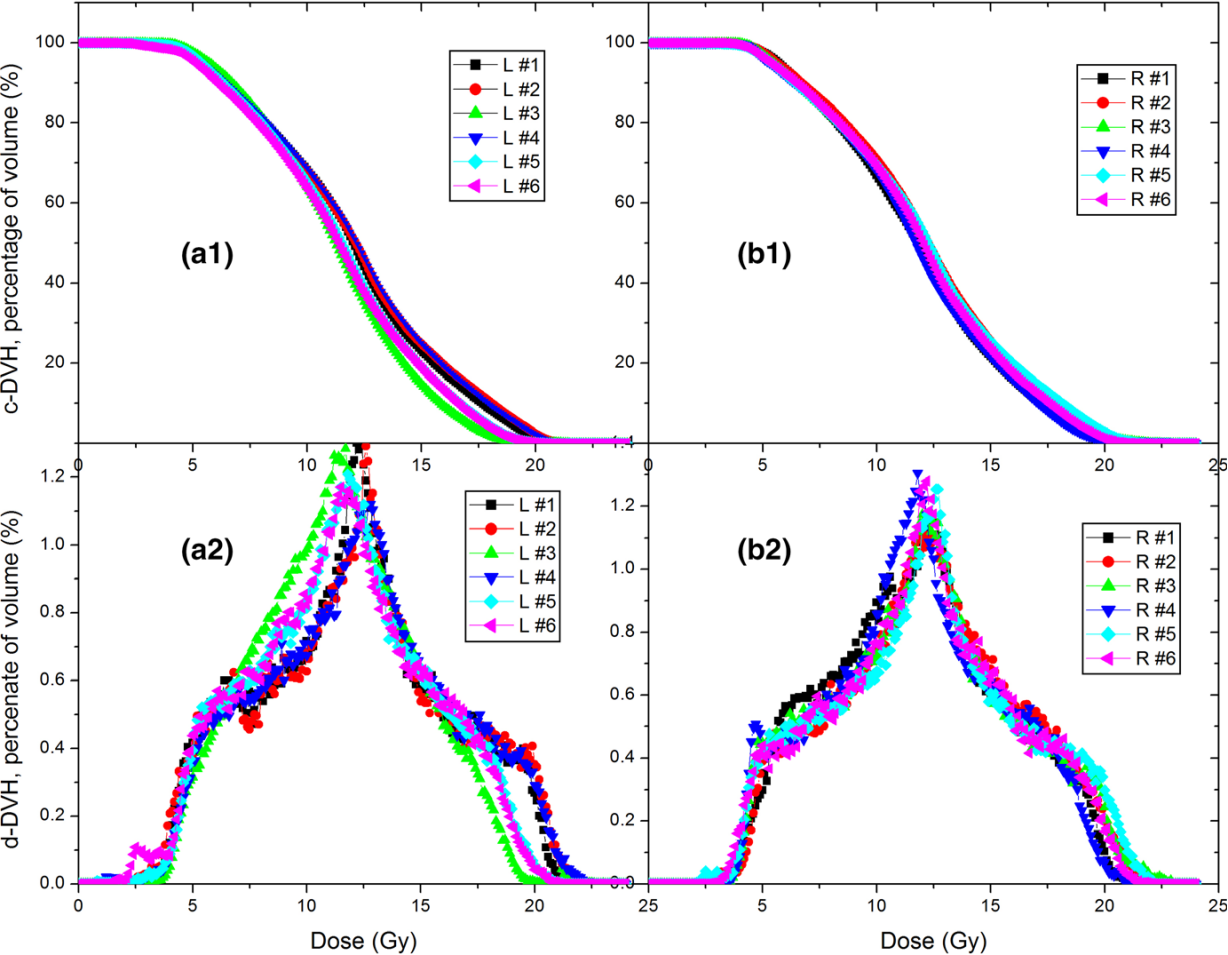
Dose‐volume histogram (DVH) curves of six left and six right breast GRID therapy cases. Upper panel a1 and b1 are c‐DVHs, lower panel a2 and b2 are the d‐DVH curves.

Figure 4 A1 and A2 show the c‐DVH and d‐DVH (differential dose‐volume histogram) curves of PTVs of six left breast GRID plans, B1 and B2 are for six right breast GRID plans. As shown in the figures, all 12 MLC‐based GRID therapy plans are very similar regardless of their breast sides and the sizes of target volumes. The dosimetric metrics of all plans were extracted and listed in the tables. (Tables 2 and 3).

**Table 2:** Volume, V100, V90, V70, V50, and V20 of planning target volume for left and right breast GRID plans.

Left case #	Volume (cc)	V100 (%)	V90 (%)	V50 (%)	V20 (%)
	**Left breast**				
L1	432	1.21%	8.54%	68.23%	99.63%
L2	354	2.42%	10.40%	68.12%	99.01%
L3	1778	0.19%	1.91%	64.06%	99.77%
L4	556	2.29%	9.69%	68.73%	99.26%
L5	954	0.23%	4.36%	65.16%	99.34%
L6	891	0.21%	3.86%	63.95%	98.32%
Average	828	1.09%	6.46%	66.38%	99.22%
Right case #	**Right breast**				
R1	746	0.44%	6.45%	66.90%	99.68%
R2	1045	1.92%	8.88%	70.93%	99.58%
R3	562	2.11%	9.11%	68.93%	99.87%
R4	1173	1.00%	5.86%	68.06%	99.73%
R5	724	2.76%	10.61%	69.52%	99.44%
R6	553	1.21%	8.08%	69.30%	99.42%
Average	801	1.57%	8.17%	68.94%	99.62%

V100, V90, V50, and V20 represent the percentage of PTV volume covered by 100%, 90%, 50%, and 20% of prescription dose, respectively.

**Table 3:** Dosimetric parameters of PTV of left and right breast GRID plans.

	Mean (Gy)	Max dose (Gy)	D90 (Gy)	D50 (Gy)	D20 (Gy)	D10 (Gy)	D5 (Gy)	Low dose constraint	PVDR (D10/D90)
Left case #	**Left breast**								
L1	12.05	22.23	6.35	12.05	15.55	17.65	18.9	96.87%	2.78
L2	12.16	22.55	6.2	12.1	15.9	18.1	19.3	96.13%	2.92
L3	11.34	21.05	6.75	11.25	14.2	15.8	16.9	97.88%	2.34
L4	12.18	23.24	6.25	12.05	15.8	17.8	19.1	95.99%	2.85
L5	11.58	22.21	6.25	11.4	14.75	16.65	17.75	96.33%	2.66
L6	11.45	22.07	6.15	11.45	14.8	16.6	17.7	95.82%	2.70
Average	11.79	22.23	6.33	11.72	15.17	17.10	18.28	96.50%	2.70
Right case #	**Right breast**								
R1	11.82	21.85	6.55	11.75	15.2	17.1	18.25	97.67%	2.61
R2	12.28	23.91	6.65	12.2	15.8	17.65	18.85	97.72%	2.65
R3	12.09	23.76	6.25	11.9	15.55	17.6	18.9	96.15%	2.82
R4	11.72	22.47	6.3	11.6	15.1	16.9	17.95	96.18%	2.68
R5	12.22	23.33	6.3	12.15	15.9	18	19.25	96.46%	2.86
R6	12.02	22.36	6.25	11.8	15.3	17.35	18.5	95.50%	2.78
Average	12.03	22.95	6.38	11.90	15.48	17.43	18.62	96.61%	2.73

Max dose: Dose received by 0.01 cc of PTV volume. D90: Dose covering 90% of PTV volume. Low dose constraint: 5 Gy or more should be given to the 95% of PTV volume.

Table 2 indicates that the 100% isodose line (20 Gy) only covered <3% of PTV, 50% of isodose (10 Gy) covered more than 63% of PTV, and 20% of prescription isodose line (4 Gy) will cover more than 98% of PTV.

Table 3 shows the dose heterogeneity metrics of PTV. From the results of all GRID plans, D90, D50, and D10 (isodose covering 90%, 50%, and 10% of target volume), respectively, were 6.35, 11.81, and 17.26 Gy. The average mean dose was 11.91 Gy.

The ratio of D10/D90, which is envisioned to replace the traditional PVDR, is found in the range between 2.34 and 2.92. Average PVDR is about 2.72. (Table 3).

### 3.B Doses to the OARS

Because the patients were treated in prone position, the dose received by lung and heart is minimal. The average mean dose of left lung was just 0.17 Gy for left breast plans, and the average mean dose of right lung was just 0.33 Gy for right breast plans. The average mean dose received by contralateral lung was found to be close to zero. V5Gy was 0.15% for left lung and 1.25% for right lung, respectively. The average mean heart dose was only 0.27 Gy. The average maximum dose of rib was 15.3 Gy, average mean dose of rib was 4.27 Gy. (Table 4).

**Table 4:** Maximum and mean doses of heart and ribs.

	Lung V5 Gy (%)	Lung Mean dose (Gy)	Heart Mean dose (Gy)	Rib Max dose (Gy)	Rib Mean dose (Gy)
**Left case #**	**Left breast**				
L1	0.04	0.1	0.27	16.48	3.62
L2	0.4	0.2	0.24	10.08	3.49
L3	0.01	0.16	0.47	18.74	5.08
L4	0.02	0.09	0.31	19.75	4.39
L5	0.4	0.38	0.68	17.02	4.41
L6	0	0.09	0.22	8.54	1.66
Average	0.15	0.17	0.37	15.10	3.77
**Right case #**	**Right breast**				
R1	0	0.16	0.11	6.25	0.98
R2	2.2	0.50	0.18	18.43	7.43
R3	2.6	0.56	0.28	18.97	8.25
R4	0.07	0.14	0.16	13.14	2.93
R5	1.8	0.39	0.21	16.42	5.59
R6	0.8	0.22	0.08	19.94	3.46
Average	1.25	0.33	0.17	15.53	4.77

Max dose: Dose received by 0.01 cc of concerned volume.

### 3.C EUDs, TRs, and MUs

The Table 5 shows the TRs, EUDs, and MUs of 12 GRID plans. It shows the TR of C1 is greater than TR of C2 for the same type of interspersed normal tissue N1. The EUDs derived from the MLQ model were found to differ by 2% between two types of breast cancer cells. From Niemierko’s equation, the EUDs differ by 4% for the same two types of cancer cells.

**Table 5:** TRs, EUDs and MUs for left and right breast GRID plans.

Case #	TR (C1/N1)	TR (C2/N1)	EUD (Gy) C1 (MLQ)	EUD (Gy) C2 (MLQ)	EUD (Gy) C1 (Niemierko)	EUD (Gy) C2 (Niemierko)	MUs
Left breast							
L1	9.61	7.53	8.83	8.67	9.45	9.78	2557
L2	10.54	8.33	8.69	8.54	9.34	9.69	2603
L3	9.53	7.48	8.92	8.77	9.46	9.71	2403
L4	13.92	10.94	8.83	8.68	9.46	9.85	2681
L5	11.03	8.76	8.69	8.54	9.27	9.58	2601
L6	12.08	9.72	8.39	8.25	9.00	9.33	2567
Average	11.12	8.79	8.73	8.58	9.33	9.66	2569
Right breast							
R1	9.76	7.66	8.90	8.75	9.48	9.78	2636
R2	12.08	9.28	9.04	8.87	9.69	10.03	2680
R3	9.62	7.52	8.88	8.73	9.51	9.83	2730
R4	9.80	7.69	8.77	8.61	9.37	9.68	2586
R5	11.27	8.84	8.79	8.63	9.44	9.79	2605
R6	10.66	8.30	8.80	8.64	9.44	9.77	2526
Average	10.53	8.22	8.86	8.71	9.49	9.81	2627

TR (C1/N1): if the radiosensitive normal tissue N1 was interspersed in the breast cancer C1. TR (C2/N1): if the radiosensitive normal tissue N1 was interspersed in the breast cancer C2.

The total MUs of each GRID plan range from 2403 to 2730, this is a practical range considering a prescription of 20 Gy will be delivered. When a dose rate of 600 MU/min was used, our experimental verification found each plan needs about 15 min to deliver four fields of treatment including setup and image verification time.

Of the note, the traditional cerrobend GRID collimator is used to treat breast patient only in supine position with the gantry angle equal or close to 0 degree, this is mainly for the patient safety reason. Because a heavy GRID block collimator (up to 22 kg) is deemed to be unsafe, if it is mounted in the gantry tray holder at a large oblique angle. Consequently, the high dose beamlets of GRID field will penetrate part of heart and lungs in their paths.

In addition, because the GRID field of cerrobend GRID collimator is visually verified from the breast surface, so the imaging devices are not involved. In an instance of cerrobend GRID radiation therapy treatment planning, the GRID field was found to have an output factor 0.89 cGy/MU at the depth of 6 MV beam d*max* of GRID field central hole, it was calculated that 2247 MUs would be needed for giving 20 Gy at the d*max* depth of the GRID field (2000 cGy/0.89 cGy/MU). If the machine dose rate is 600 cGy/min, the treatment will take just 3.75 min. The treatment time of using cerrobend GRID collimator is shorter than that of using MLC‐based GRID techniques. The major drawbacks of using cerrobend GRID collimator are unknown organ doses and poor conformality to the target volume.

Per the literature,^7^, ^10^ at 20 Gy prescription dose, the GRID radiation therapy with cerrobend GRID collimator can reach EUD ~ 4.2 Gy, TR ~ 2.5, and PVDR ~ 4.9. MLC‐based GRID plans of this study have a greater EUD (~8.7 Gy) and TR (~9.0), but a smaller PVDR (2.7).

### 3.D Dosimetric metrics of 2‐cm aperture GRID plans

**Fig. 5:**
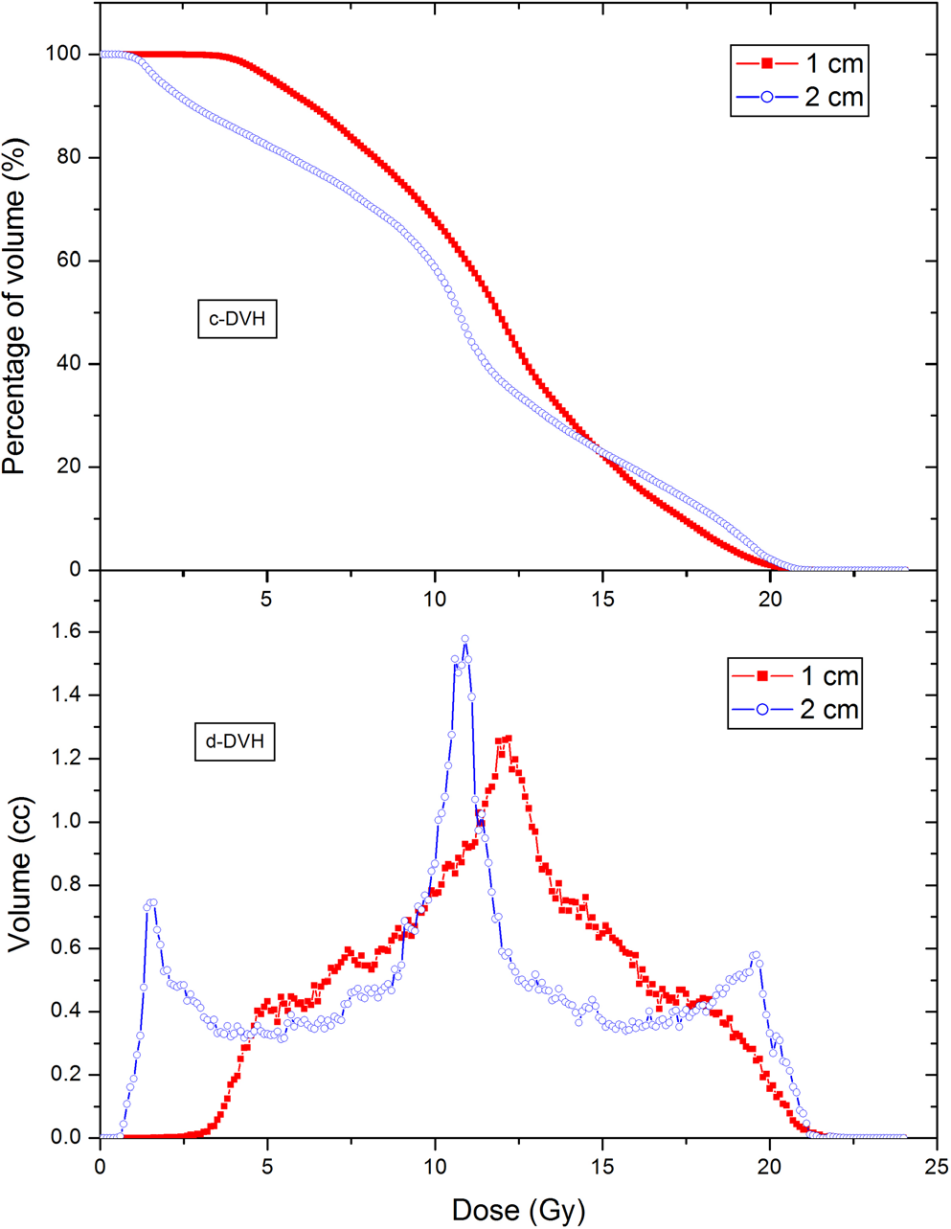
A Dose‐volume histogram comparison between 1 and 2‐cm aperture GRID therapy plans for the same patient.

**Fig. 6:**
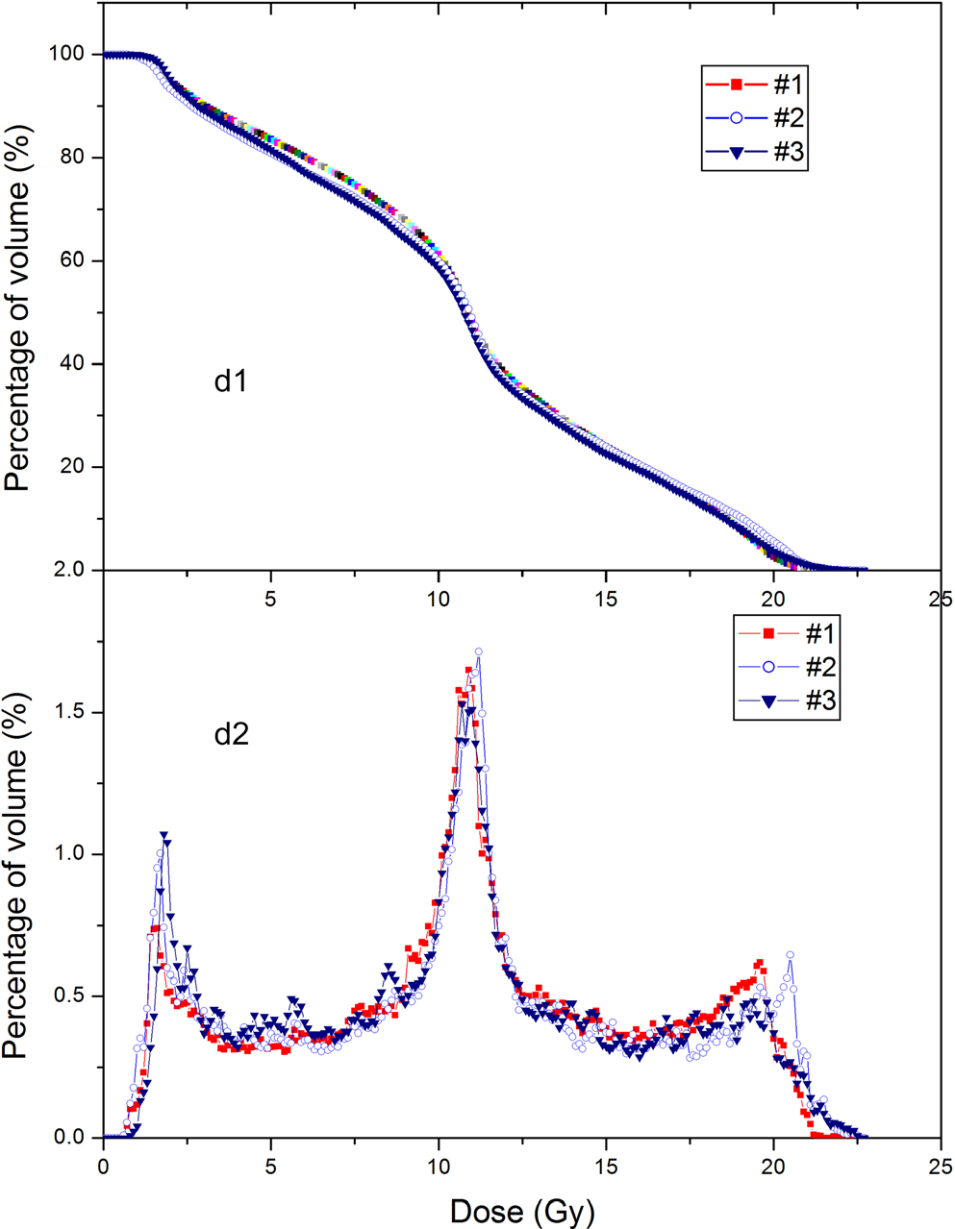
Dose‐volume histogram (DVH) curves of three 2‐cm aperture GRID therapy plans. d1 is the c‐DVH curves, d2 is the d‐DVH curves.

Figure 5 shows a comparison between 1‐cm and 2‐cm aperture GRID therapy plans for the same patient. Figure 6 shows the c‐DVH and d‐DVH curves of 2‐cm aperture GRID plans of three patients. Table 5 shows the results of the TRs, EUDs, PVDRs, and MUs from 2‐cm aperture plans compared with 1‐cm aperture GRID plans for the same patients. The 2‐cm aperture plans were found to have a smaller TR, EUD, and MU but greater PVDRs than their 1‐cm counterparts (Table 6).

**Table 6:** TRs, EUDs, and PVDRs of 1‐cm and 2‐cm aperture GRID plans for three randomly picked patients. Breast cancer cell line C1 and radiosensitive normal tissue N1 are used in TR calculations.

Case #	1‐cm aperture					2‐cm aperture				
	tr (c1/n1)	EUD (Gy) (MLQ)	EUD (Gy) (Niemierko)	PVDR	MU	TR (C1/N1)	EUD (Gy) (MLQ)	EUD (Gy) (Nemierko)	PVDR	MU
1	12.08	9.04	9.69	2.65	2680	5.72	6.09	6.57	6.63	2246
2	11.27	8.79	9.44	2.86	2605	5.63	5.94	6.40	7.21	2255
3	10.66	8.80	9.44	2.78	2526	6.60	6.24	6.77	6.1	2181
AVG	11.34	8.88	9.52	2.76	2604	5.98	6.09	6.58	*6.65*	*2227*

## 4 DISCUSSIONS

Spatially fractionated GRID radiation therapy has primarily been used for debulking large tumors or for increasing radio‐immuno response in order to arrange an effective conventional treatment or for palliative treatment. To date, ample clinical evidence has accumulated for the high symptomatic and clinical response and minimal toxicity of GRID therapy in palliatively and definitively treated tumors with excessive bulk and/or therapy resistance.^1^, ^2^, ^3^, ^4^, ^5^, ^33^

EUD is an important parameter for assessing GRID therapy plan. EUDs calculated by Niemierko’s equation were found to be greater than that obtained from the MLQ model. This is understandable because, when the killing of 2 Gy is extended to the dose >10 Gy, the killing rate will be overestimated because of neglected cell repair which is more important at high dose range,^19^ this resulted in the overestimation of EUD. In MLQ, however, the overly predicted killing by LQ model in high dose range was corrected by introducing an empirical shifting factor, δ. Therefore, it was found if Niemierko’s equation had to be used in assessing GRID plans made by this approach, EUDs would be overestimated by 7% (for acutely responding breast cancer C1) and 13% (for slow responding C2) comparing with that calculated by the MLQ model.

Comparing the 1‐cm aperture and 2‐cm aperture GRID therapy plans, some obvious differences were seen: (a) c‐DVH curve of PTV moves to the lower doze zone, but the high dose zone gets bigger when the aperture size was increased from 1 to 2 cm; (b).The 1‐cm plans showed only one peak, but 2‐cm plans had three peaks in d‐DVH curve. In the 1‐cm plans, the volumes receiving small dose and high dose are relatively small, the majority of volume receives around half of prescription dose. The three peaks of d‐DVH curve indicated that, in the 2‐cm plans a significant portion of the target volume gets half of prescription dose, a significant portion of the volume gets a large dose (~18 Gy), and another significant portion of the volume gets a small dose (~3 Gy). The average EUD and TR of 2‐cm plans are 37% (6.09 vs 8.88 Gy) and 90 % (5.98 vs 11.34) smaller than that of 1‐cm plans. Because a larger EUD means a larger possibility of tumor control and a larger TR means a larger portion of normal tissue sparing comparing to the open‐field radiotherapy and thus less possibility of radiation complication, the results may have implied that the 1‐cm GRID plans are superior over 2‐cm ones in theory, although this needs to be verified by clinical data. In addition, the PVDRs of three 2‐cm plans are greater than that of the 1‐cm GRID plans by a factor of 2.41, this is because the 2‐cm plan quadrupled the shielded area and open area as well, and thus the shielded low dose zones will get less dose from internal scattering and the volume of hot dose zone will be increased. The role played by the PVDR in the GRID therapy needs to be examined by a systematic clinical trial.

Both the 1 and 2‐cm plans showed to have a practical range of MUs. Average number of MUs of 1‐cm plans is 2598 MUs, roughly each field has about 650 MU. For a 2‐cm plan, the average MU is 2227, each field has about 560 MU. This indicated the treatment can be delivered in short time, so the concern of patient discomfort and organ and target movement during the treatment can be alleviated.

Recently, Costlow et al.^11^ explored MLC‐based GRID therapy techniques by using bulky lung tumor as target and creating Electronic compensation Tubes (Ecomp‐tubes), Ecomp‐Circles, Ecomp‐Squares, Ecomp‐Weave, IMRT, and IMRT‐Weave six different plans for each patient, demonstrated the versatility of MLC and modern TPS for making diverse GRID therapy plans. Parkhrel et al.^15^ reported a study in which they employed an onboard MLC to generate the three dimensional (3D) spatially fractionated radiation therapy plans for treating deeply seated tumors at diverse anatomic sites. Parkhrel used 6 coplanar gantry angles in every plan and a pair of MLC formed strip‐like fields for each angle. Similar to our approach, at each gantry angle the GRID field was formed by rotating second field collimator angle 90°, thus a 3D lattice radiotherapy plan can be generated. In another recently published study Kopchick et al.^16^ demonstrated that, a stereotactic radiotherapy apparatus which was initially designed for making breast SBRT, can be used to make a breast lattice radiotherapy therapy plan as well. The dosimetric parameters of aforementioned plans are similar to our plans. The advantages of our dosimetric approach are: (a) the patient anatomic structures are maximally protected from the high dose strips of grid fields by using a prone position, (b) no need to purchase an additional apparatus, (c) it can accurately report the doses to the target volume and OARs (Tables 2, 3, and 4), and (d) it also can reduce the OAR doses via adjusting the beam shapes, orientations, and intensity, the dosimetric data can ensure that the plan is safe and can achieve similar and even potentially better treatment outcomes because of closely tailoring the target dose and normal structure considerations. With our presented dosimetric approach that uses two gantry angles, multiple beams, prone position, and modern radiotherapy TPS, the toxicities are expected to be less than the treatments using the Cerrobend GRID block with single angry angle, single beam, supine position, and no consideration for target and OAR geometries.

## 5 CONCLUSIONS

GRID therapy for breast cancer is feasible with a modern external beam TPS and onboard multileaf collimator (MLC). The plans generated by MLC and TPS in the prone position showed consistent dosimetric quality results and a strong advantage in accommodating targets and maximally sparing normal structures. When considering different radioresponses of cancerous cells of breast, GRID therapy with 1‐cm aperture at the 20 Gy prescription dose was found to have an EUD ranging from 8 to 10 Gy. The PVDR is from 2.34 to 2.92. GRID therapy can also spare more than nine times more radiosensitive normal cells comparing with the open‐field radiation boost at the same cancer cell killing. When the aperture size is increased from 1 to 2 cm, EUD and TR decreased, but the PVDR increased.
